# Confucian Familism and Shared Decision Making in End-of-Life Care for Patients with Advanced Cancers

**DOI:** 10.3390/ijerph191610071

**Published:** 2022-08-15

**Authors:** Yuexi Yang, Tingting Qu, Jinyue Yang, Ben Ma, Anli Leng

**Affiliations:** 1 School of Political Science and Public Administration, Shandong University, Qingdao 266237, China; 2 Lumanity, Las Vegas, NV 89135, USA; 3 Qingdao Center for Women and Children’s Health and Family Planning Service, Qingdao 266034, China

**Keywords:** Confucian familism, shared decision-making, end-of-life care, advanced cancers, China

## Abstract

Shared decision-making (SDM) has been institutionally recognized as clinically effective by many Western healthcare systems. Nevertheless, it appears culturally unattractive in China, a country that adheres to Confucian familism which strongly prefers collective family decisions. This study examined this conflict and assessed the influence of Confucian familism on SDM in end-of-life (EOL) care for advanced cancer patients. Between August and November 2018, 188 EOL advanced-cancer patients were randomly recruited from 640 cancer hospital medical records at a Tertiary A-level hospital in Shandong province. Eventually, 164 (87.23%) sample patients were included in the statistical analysis after the non-responsive cases (4.79%) and missing value (7.98%) were removed. SDM was measured through SDM-Q-9, and the patient’s siblings were used as indicators of Confucian Familism. Of the 164 patients, the mean SDM score was 38/100; 47.6% were thoroughly unfamiliar with their treatment plans and fell outside the decision-making procedure. Each patient had four siblings on average. Ceteris paribus, more siblings led to lower SDM. Moreover, being 56–65 years old and open-minded were associated with higher SDM, while higher satisfaction of the quality of EOL care yielded lower SDM. In conclusion, Confucian familism weakened patient–clinician SDM in EOL care for advanced cancer patients.

## 1. Introduction

Shared decision-making (SDM) is a clinical model that allows patients or their agents to negotiate medical care and make health care decisions with their clinicians. The model takes into account both the knowledge, experience, and evidence-based diagnoses of healthcare professionals and the values, goals, and preferences of patients [1]. It is derived from Western socio-economic culture, which is founded on individualism, breaking up “power asymmetry”, and navigating the middle ground between “daddy knows best” paternalism and rampant consumerism [2,3]. SDM has demonstrated to be effective in clinical practice [4] and has been institutionally recognized by healthcare systems in many countries. For example, the United States added SDM to The Patient Protection and Affordable Care Act in 2010 [5], and Switzerland created decision aids and specific SDM training programs at medical schools [6].

Because China leads the world in cancer incidence and mortality, it is also supposed to embrace the enormous clinical benefits that come from SDM. In 2020, China accounted for 4,568,754 of 19,292,789 new cancer cases (23.7%) and 3,002,899 of 9,958,133 cancer deaths (30.4%) [7,8]. Nevertheless, SDM seems culturally unappealing for a country deeply rooted in Confucian familism for over 2000 years. In fact, Confucian familism strongly favors family-centered collective decisions, which could violate the principles of SDM between patient and clinician [9]. Therefore, shying away from SDM may lead to much higher opportunity costs than in Western countries.

Confucianism is a Chinese ethical tradition that also structures the cultural foundation of Japan and South Korea and Southeast Asian countries such as Singapore, the Philippines, and Vietnam [10]. Confucian familism insists that family as a whole rather than any individual member has ontological priority and that collective decisions address matters of crucial concern [10]. Specifically, it empowers senior family members (parents and elder brothers and sisters) to make decisions on behalf of younger ones. The more that family decision-making agents believe they are acting in the best interests of their principals, the less autonomy the principals have. 

This principal–agent problem severely undermines the SDM mechanism between patient and clinician in end-of-life (EOL) care. In China, it is a social norm for an oncologist to discuss progress and treatment options with a family agent instead of the patient [11,12]. On one hand, the agent seems reluctant to tell the patient the true state a disease to help him or her stay positive [13]. On the other hand, driven by filial piety in Chinese culture [14], the agent tends to choose rather aggressive options for maximum effect regardless of any physical or psychological side effects. However, without such an agent authorized by Confucian familism, the patient is likely to prefer more conservative treatments to maintain quality of life (QoL).

Despite China’s urgent need to tackle this cultural obstacle to develop patient–clinician SDM, relevant evidence is rather sparse. With only a few qualitative studies, it is not clear how much SDM for cancer patients there is. It is also unknown how and to what extent Confucian familism constrains the efficacy of SDM in end-of-life (EOL) care.

To fill in the literature gap, the following three research questions were addressed in this study.

1. How to measure SDM in EOL care for patients with advanced cancers?
2. What is the impact of Confucian familism on SDM in EOL care?
3. What are other factors that may influence SDM in EOL care?

## 2. Materials and Methods

### 2.1. Participants

**Figure 1:**
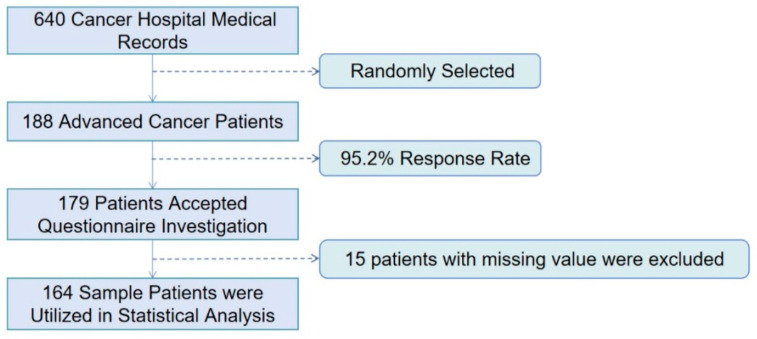
Flow chart illustrating inclusion of survey participants.

The process of recruiting the participants was illustrated in Figure 1.

Between August and November 2018, 188 EOL advanced cancer patients were randomly recruited from 640 cancer hospital medical records at a Tertiary A-level hospital in Shandong, China. To be included a patient had to have no cognitive impairments. Prior studies found that a patient’s decision-making can be projection biased; that is patients with severe diseases prefer to make decisions based on current severity [15]: the worse the health status, the more stable the preference [16]. Therefore, three selection criteria were added: patients had to be diagnosed with Stage 3 or 4 cancer; hospitalized receiving therapy, such as surgery, radiotherapy, chemotherapy, or targeted therapy; and at least 44 years old. Compared to children and adolescents, older people predict emotional responses more accurately and preserve more stable preferences [17]. 

The survey was conducted face-to-face by professional interviewers, leaving a sample of 179 patients and a 95.2% response rate. All interviewees gave informed consent and answered all questions without external assistance. Fifteen patients were excluded after responses were sorted out and missing values were removed. In the end, 164 (87.23%) sample patients were included.

### 2.2. Variables

#### 2.2.1. Measuring SDM

SDM-Q-9, a patient reporting measurement method, was applied to collect patient information in this study. Originating in Germany, the scale is based on Elwyn’s model of competences for involving patients, as well as the Ottawa Decision Support Framework [18]. A 24-item version was first summarized using the Delphi method and then refined by a 9-item version [19]. Tested by using 2351 primary care patients from Germany, the results showed a high reliability with a high internal consistency (Cronbach’s α > 0.9) and item discrimination, as well as higher face and factor validity [19]. Then SDM-Q-9 was gradually translated into other languages for international research: first into the Western languages such as English [20], Dutch [21], Spanish [22], and Hungarian [23] and then into Chinese [24], Japanese [25], and Malay [26], which are spoken in countries of Confucian familism. Sample patients were recruited locally, and the questionnaires in different languages all showed adequate reliability and acceptable validity parameters. Therefore, SDM-Q-9 was demonstrated to be accountable, reliable and viable to be applied in this study. The 9 items comprised the following:

1. My doctor made clear that a decision needs to be made.
2. My doctor wanted to know exactly how I want to be involved in making the decision.
3. My doctor told me that there are different options for treating my medical condition.
4. My doctor precisely explained the advantages and disadvantages of the treatment options.
5. My doctor helped me understand all the information.
6. My doctor asked me which treatment option I prefer.
7. My doctor and I thoroughly weighed the different treatment options.
8. My doctor and I selected a treatment option together.
9. My doctor and I reached an agreement on how to proceed.

To access SDM for each item, a patient was asked to self-report on a scale from 0 to 5: “Completely Disagree”, “Strongly Disagree”, “Somewhat Disagree”, “Somewhat Agree”, “Strongly Agree” or “Completely Agree”. To measure SDM across all items, a numeric value for each item was added to produce a total score ranging from 0 to 45. By multiplying by 20/9, the raw range (0, 45) was leveled up to ([0, 100), in which 0 and 100 represented no SDM and full SDM, respectively. It was more intuitive to interpret the result by the new range: the closer to 100, the higher a patient’s perception of SDM and the stronger sense of autonomy throughout the clinical decision-making process. 

#### 2.2.2. Measuring Confucian Familism

To quantify Confucian familism, a patient was asked about the number of siblings. Little research has been published about the influence of sibling numbers on SDM. Only a study targeting youth found that sibling numbers had no effect on decision-making power [27]. The idea to advocate reproduction is rooted throughout the Confucianism [28]. Therefore, in this study, patient’s sibling numbers were used as a proxy to measure Confucian familism. The more siblings, the higher likelihood that family members would claim to be decision-making agents on behalf of the patient. Note that parents are believed to be the most powerful and respectful family members for decision-making and often step in to communicate with clinicians on behalf of their children [29]. Unfortunately, most of the sample patients’ parents had died before the survey began. Therefore, it was assumed that there were no parent decision-making agents in this study. 

#### 2.2.3. Other Variables

Following a standard medical questionnaire, a patient’s baseline characteristics were collected including demographic information like age and gender; socioeconomic information such as education, marital status, residence (rural or urban), and head of the household or not, and monthly income; and disease information like cancer type and stage.

Second, a patient’s personality was drawn using the Big Five Inventory-10 (BFI-10) [30]. Some scholars empirically verified how personality characteristics influenced a patient’s decision-making style [31,32]. However, the relationship between patient personality and SDM level among cancer patients in EOL care was hardly examined. BFI-10 consisted of five dimensions: openness to experiences, conscientiousness, extraversion, agreeableness and neuroticism. Each dimension was measured based on a negative and then a positive description. For example, regarding neuroticism, the patient was asked “I see myself as someone who is relaxed and handles stress well” first and then “I see myself as someone who gets nervous easily”. A patient was asked to choose 1, 2, 3, 4 or 5, with 1 being “Strongly Agree” and 5 being “Strongly Disagree” for a negative description but 1 being “Strongly Disagree” and 5 being “Strongly Agree” for a positive description. The average score of both descriptions was taken to measure neuroticism. The higher the average for each dimension, the more one is open, conscientious, extraneous, agreeable or neurotic. 

Third, a patient was asked to self-access the EOL care received at the hospital. Several studies took SDM as an independent variable to explore its positive influence on cancer patients’ satisfaction [33,34]. In this study, satisfaction was treated as independent variable and refined into how much one (dis)likes the clinician specialty, staff approachableness, service quality, service affordability, and service accessibility (i.e., the distance between one’s residence to the hospital). For each question, a patient chose 1, 2, 3, 4 or 5 with 1 being “Strongly Dislike” and 5 being “Strongly Like”. The chosen score was used to measure each of the five aspects without further adjustment. 

Finally, QoL was thought to correlate positively with SDM approaches in previous research [35]. In this study, QoL was measured by one representative comprehensive question from the McGill QoL Questionnaire. A patient was asked to self-evaluate QoL score considering the physical, emotional, social, spiritual, and financial parts of his or her life over the previous two days [36]. An interviewee chose an integer between 1 to 10 with 1 being “Very Bad” and 10 being “Very Good”. The score was used to measure QoL without further adjustment. 

### 2.3. Statistical Analysis

A series of univariate ordinal logistic regressions was conducted first to explore the association between the overall SDM score and all testable factors such as a patient’s siblings, age, gender, socioeconomic status, personality, EOL care assessment and QoL. Based on the traditional stopping rule in previous literature, factors with a threshold *p* < 0.2 were selected as explanatory variables [37]. 

Next, multivariate-ordered logistic regression was explored. Before the regression, intervals (0–33), (33–66) and (66–100) were used to categorize the SDM level into low, moderate and high, respectively. The regression results were reported in form of an odds ratio (OR), the significance of which was tested at 5%. STATA 14 was adopted to perform the analysis.

## 3. Results

### 3.1. Descriptive Statistics of SDM

**Figure 2:**
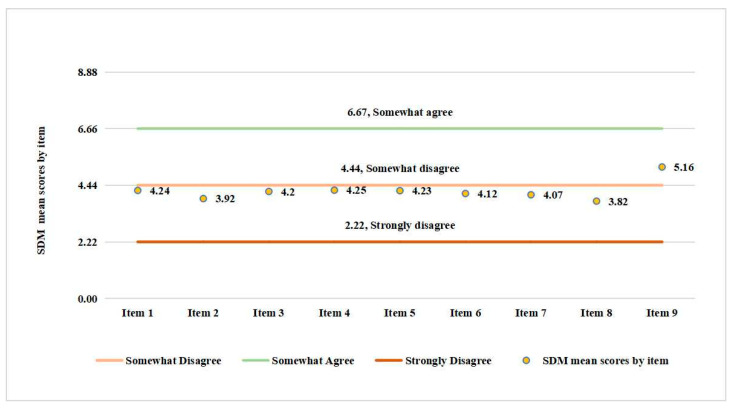
SDM-Q-9 mean scores by item.

Of the 164 sample patients, the mean SDM score per item is reported in Figure 2.

For each item, the score was interpreted based on 0, 2.22, 4.44, 6.67, 8.89 and 11.11 meaning “Completely Disagree”, “Strongly Disagree”, “Somewhat Disagree”, “Somewhat Agree”, “Strongly Agree” and “Completely Agree”, respectively. Of all the 9 items, item 8 had the smallest mean (3.82), indicating that on average, a patient tended to strongly disagree with the statement “My doctor and I selected a treatment option together”. Item 9 had the largest mean (5.16), indicating that on average, a patient almost disagreed with the statement “My doctor and I reached an agreement on how to proceed”. The results implied that patients received treatments not decided between themselves and their physicians. Patient distribution for each item based on SDM-Q-9 is reported in Appendix A Table A1.

**Figure 3:**
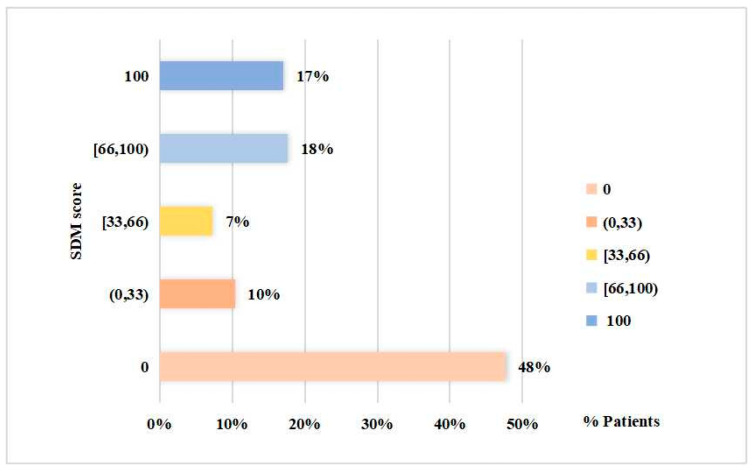
SDM-Q-9 scores distribution.

Furthermore, the mean (median) of the overall SDM was 38 (11.11). Recalling that 40, 20 and 0 indicated “Somewhat Disagree”, “Strongly Disagree” and “Completely Disagree”, respectively, the results suggested that SDM was rather low on average and half of the patients completely or strongly disagreed that they perceived SDM. Next, the distribution of the overall SDM is presented in Figure 3.

Specifically, 78 and 28 patients scored 0 and 100, respectively; the former completely disagreed with all items while the latter completely agreed. In other words, 48% of patients were completely unaware of their treatment plans at any level and were not engaged throughout the entire SDM procedure; 17% perceived or understood their treatment plans very well and were fully involved in the whole SDM process. 

### 3.2. Confucian Familism Associated with SDM

**Figure 4:**
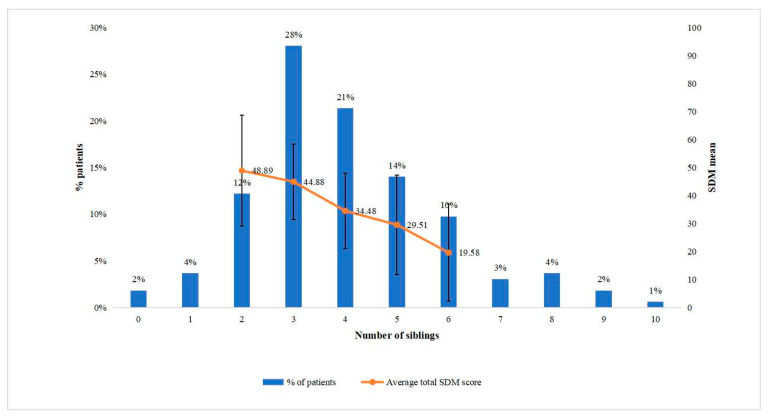
SDM scores and number of siblings. Notes: 95% confidence intervals were not available when the percentage of patients was lower than 5.

The distribution of number of siblings is summarized in Figure 4. 

Among the 164 sample patients, 2% patients did not have any siblings; 44% had 1 to 3; and 54% had four or more. The results suggested that almost all patients had at least one potential decision-making family agent for their EOL care options. Note that SDM scores decreased from 48.89 to 19.58 as the number of siblings increased from 2 to 6 (Figure 4). It implied that SDM between a clinician and a patient became less likely when Confucian familism became more likely.

### 3.3. Other Factors Associated with SDM

Number of patients (%) for baseline characteristics are summarized in Table 1.

The patients were 61.57 years old on average; 69.51% were males; 67.68% received at least six years of education; 93.29% were married; 62.2% resided in rural areas; 71.95% registered themselves as head of the household; 73.17% reported monthly income below $454. Furthermore, the sample patients showed good variation across cancer types and stages: 42.68% of cancers were urological (renal, bladder, and prostatic); 28.66% were digestive (gastric, colorectal, esophageal, pancreatic, and liver); and 17.07% were respiratory (lung, thymic); 50.61% were in Stage 3.

**Figure A1:**
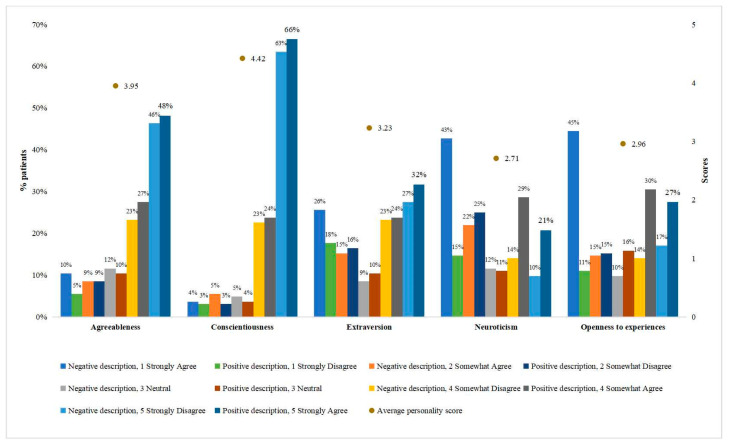
Personality of sample patients based on BFI-10 (*n* = 164). Key: BFI, Big Five Inventory.

Next, regarding patient personality, the mean score of agreeableness, conscientiousness, extraversion, neuroticism and openness to experiences was 3.95, 4.42, 3.23, 2.71, 2.96, respectively (see Appendix B, Figure A1). With 3 being “Neutral”, it was implied that the patients were strongly agreeable and conscientious, neurotic and open to experiences, but neutral regarding extraversion.

**Figure A2:**
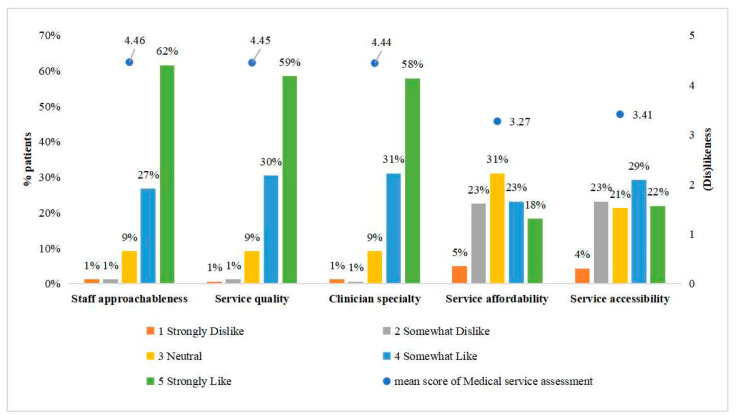
Medical service assessment of sample patients (*n* = 164).

Third, regarding the EOL care assessment, staff approachableness, service quality, and clinician specialty were completely or strongly liked by nearly 90% patients: their means were 4.46, 4.45, and 4.44, respectively (see Appendix B, Figure A2). With 1 being “Strongly Dislike” and 5 being “Strongly Like”, the results implied that patients were highly satisfied with the quality of care. However, patients were almost neutral with respect to affordability (mean, 3.27) and accessibility (mean, 3.41), and about 30% strongly or somewhat disliked their service affordability and accessibility. 

**Figure A3:**
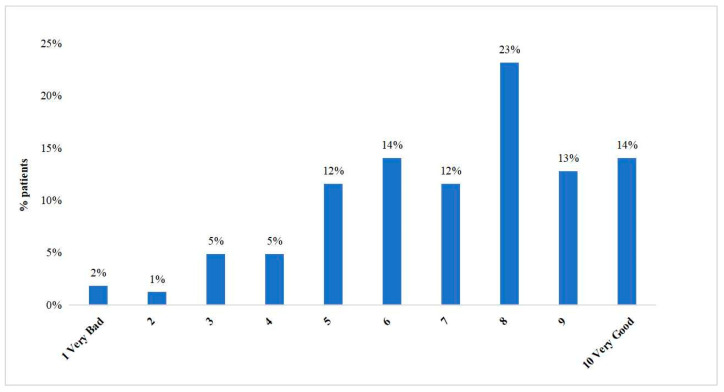
QoL of sample patients (*n* = 164). Key: QoL, quality of life.

Finally, the mean (SD) of self-evaluated QoL was 7.02 (2.2) (see Appendix B, Figure A3). Only 2% rated their QoL as very bad. With 10 being “Very Good”, the results suggested that in the last two days before the survey the patients assessed themselves as a achieving a good physical, emotional, social, spiritual, and economic QoL.

### 3.4. Regression Results

The univariate analysis results indicated that 7 factors were significant at 20% level including Confucian Familism (i.e., number of siblings), age, gender, household head or not, satisfaction with medical service, extraversion, and openness (see Appendix A Table A2). The qualified set of candidate variables were entered into multivariate ordered logistic regression and the results were presented in Table 2. 

Model 4 ran regression on all of the 7 explanatory variables and passed the parallelism test (See Appendix A Table A3). Ceteris paribus, patients with one more sibling had 0.78 lower odds of SDM being high vs moderate/low (OR = 0.78, *p* = 0.01). Also, the estimated OR was robust to different sets of explanatory variables, as reported in Model 1, 2 and 3. In addition, compared with 44–55 years old patients, 56–65 years old patients had 4.26 (*p* = 0.01) higher odds of SDM being high versus moderate/low or SDM being high/moderate versus low; but both 66–75 and 75+ years old patients had no significant ORs. One unit increase in patients’ openness yield 2.07 higher odds of their SDM being high vs moderate/low (OR = 2.07, *p* < 0.001). But, one unit increase in patients’ medical service satisfaction yield 0.53 lower odds of their SDM being high vs moderate/low (OR = 0.53, *p* = 0.01).

## 4. Discussion

This paper was the first quantitative investigation into the impact of Confucian familism on patient–clinician SDM for EOL care in patients with advanced cancers. It also considered SDM-associated factors such as age, personality and quality of EOL care.

First, Confucian familism constrain the efficacy of SDM in EOL care for patients with advanced cancer. This study showed that SDM between a clinician and patient significantly decreased with the amounts of patient siblings: The more siblings, the more likely they were to be decision-making agents on behalf of the patient. Regarding EOL care for a patient with advanced cancer, physicians often communicated with a family agent (e.g., parents or elder siblings) rather than the patient regarding progression status and treatment alternatives. Such practice violates the basic principle of SDM where clinicians should work directly with their patients concerning treatment. 

Second, higher SDM was associated with older patients. This study found that, compared with 44–55-year-old patients, 56–65-year-old patients had significantly better odds of SDM being high versus moderate or low. Note that previous studies did not reach a consensus regarding SDM among cancer patients of different ages. A scoping review summarized that most quantitative studies showed an unclear relation between age and occurrence of SDM. Moreover, some found that younger patients tended to act more proactively, while only a few reported that patients who perceived high SDM levels were significantly older than those who perceived intermediate or low SDM levels [38]. The association between age and SDM varied by cancer stage and type as well as the country or region from which the data were collected. More compressive and global studies are needed to address the ambiguous relationship between age and SDM.

Third, a higher SDM was associated with patients who were more open to clinical experiences. In fact, openness to clinical experiences was the only significant factor associated with SDM among the sampled patients. One explanation was that SDM could be quite an exotic, modern idea to Chinese patients as it originated and developed in Western societies. Cancer patients who were less open-minded preferred traditional doctor-dominant decision-making and trusted more conservative treatments. However, patient engagement in SDM can be motivated by increasing self-awareness and advocating for basic rights [39]. More actions are needed to unleash patients from old-fashioned treatment ideology to accept the new cooperative decision-making model. The imperative is to gradually educate patients and their families in treatment cognition, attitude, and behavior as well as to keep them informed of the patterns and benefits of SDM. 

Fourth, SDM was negatively associated with a patient’s degree of satisfaction with the quality of service. This study found that SDM decreased as a patient became more satisfied with EOL care, which seems inconsistent with findings in other countries. Sometimes patients were left with a sense of insecurity and vulnerability [39] as clinically experienced doctors became less sensitive to their emotional and psychological needs [40]. Moreover, SDM is more challenging, even impossible, between patients and overloaded doctors. In 2019, China had 2.2 doctors per 1000 inhabitants, whereas Austria had 5.3, ranking them 28th and 1st among 32 OECD sampled countries, respectively [41]. As expected, a fulfilling diagnosis and treatment timeframe per patient, especially for outpatients, is unrealistic in China. Patients have become more and more reluctant to trust physicians and follow their clinical instructions and decisions over the last two decades [42]. Such a trend is likely to further pull away patients from sharing decision making with their physicians. However, the negative association detected in this study was based on EOL care for patients with advanced cancer. The medical and clinical knowledge asymmetry (e.g., obscure medical terminology) between physicians and cancer patients objectively defined physicians as experts. Cancer patients who claimed to be content with their quality of EOL care not only recognized the clinical specialty of their physicians but also trusted them as sincere, rigorous and responsible caregivers. In the end, the patients retreated from SDM by leaving the majority of treatment decisions to their physicians. To promote SDM between doctors and patients, it is suggested that physicians not only encourage their patients to express their independent opinions on treatment plans and preferences, but also to treat them as co-decision makers.

Strategies to promote SDM in any country, especially those featuring Confucian familism, should be tailored to fit cultural traditions and the healthcare system. “The SDM 3 Circle Model” identified three core categories: (1) patient/family, (2) provider/team, and (3) medical context, which overlaps with the others within an “environment frame” [43]. The environmental frame includes all external, contextual factors that may influence any of the 3 circles. Therefore, under the context of Confucian Familism, implementing SDM requires the full participation of physicians, family members and patients. In view of the overall low SDM in EOL care, physicians were entrusted with more responsibility to engage cancer patients in whole medical treatment decision-making to ensure that they thoroughly weighed the different treatment options and reached a treatment agreement altogether. More specialized personnel and resources should be allocated to promote SDM. Additionally, patient decision aid (PtDA) tools could be adopted to smooth decision-making [44].

This study had several limitations. Because the survey was conducted in one province, province-level variations could not be controlled, nor could comparisons be made with a similar study in one or more urban settings. Furthermore, the cross-sectional design was not able to control the effect of time. Future research should extend it to a longitudinal study. Finally, all measures were based on self-reported values and should be further validated if comparable administrative or other real-world data become available.

## 5. Conclusions

This paper analyzed how Confucian familism constrained the efficacy of SDM. Using survey data collected from Shandong, China, the study evaluated its impact on SDM in EOL care and explored other factors associated with SDM. It was demonstrated that SDM between doctors and patients was relatively low. Furthermore, Confucian familism significantly undermined SDM in EOL care for patients with advanced cancer. Finally, lower SDM was found to be associated with younger patients, who were less open to clinicians’ experiences or were more satisfied with the quality of EOL care.

## Figures and Tables

**Table 1:** Patient baseline characteristics (*n* = 164).

Variables	*n*	%
**Age (years)**		
44–55	44	26.83
56–65	76	46.34
66–75	36	21.95
75+	8	4.88
**Gender**		
Male	114	69.51
Female	50	30.49
**Education (schooling years)**		
Low (6−)	53	32.32
Medium (7 to 9)	73	44.51
High (9+)	38	23.17
**Marital status**		
Married	153	93.29
Never married/widowed/divorced	11	6.71
**Residence**		
Rural areas	102	62.2
Urban cities	62	37.8
**Household head**		
Yes	118	71.95
No	46	28.05
**Monthly income ^1^**		
$151−	67	40.85
$152 to $454	53	32.32
$454+	44	26.83
**Cancer type**		
Respiratory	28	17.07
Digestive	47	28.66
Urological	70	42.68
Other	19	11.59
**Cancer stage**		
3	83	50.61
4	81	49.39

**Notes:**^1^ Based on exchange rate in 2018, $1.00 = ¥6.6118.

**Table 2:** Ordered Logistic Regression Results.

Factors	Model 1	Model 2	Model 3	Model 4
Number of siblings	0.83 ** (0.07)	0.81 ** (0.08)	0.80 ** (0.07)	0.78 ** (0.08)
44–55 years old (base category)				
56–65 years old	2.95 *** (1.22)	3.70 *** (1.64)	3.33 *** (1.42)	4.26 *** (1.96)
66–75 years old	2.28 * (1.14)	2.13 (1.12)	2.78 ** (1.43)	2.57 * (1.40)
75+ years old	0.47 (0.53)	0.41 (0.50)	0.33 (0.41)	0.24 (0.32)
Male (base category)				
Female	1.12 (0.70)	1.53 (0.97)	0.76 (0.50)	1.04 (0.70)
Not head of household (base category)				
Head of household	0.62 (0.40)	0.62 (0.41)	0.36 (0.25)	0.36 (0.26)
Extraversion		1.11 (0.14)		1.10 (0.14)
Openness to experiences		2.01 *** (0.37)		2.07 *** (0.39)
Service quality satisfaction			0.56 ** (0.13)	0.53 ** (0.13)
*n*	164	164	164	164

**Notes:** *** *p* < 0.01, ** *p* < 0.05, * *p* < 0.1.

## Data Availability

The data that support the findings of this study are available on request from the corresponding author.

## Appendix A. Tables

**Table A1:** Patient distribution for each item based on SDM-Q-9.

Item	Completely Disagree	Strongly Disagree	Somewhat Disagree	Somewhat Agree	Strongly Agree	Completely Agree
	*n* (%)	*n* (%)	*n* (%)	*n* (%)	*n* (%)	*n* (%)
1	94 (57.32)	2 (1.22)	1 (0.61)	7 (4.27)	12 (7.32)	48 (29.27)
2	97 (59.15)	2 (1.22)	1(0.61)	11 (6.71)	13 (7.93)	40 (24.39)
3	94 (57.32)	0 (0.00)	5(3.05)	6 (3.66)	13 (7.93)	46 (28.05)
4	94 (57.32)	2 (1.22)	2 (1.22)	6 (3.66)	10 (6.10)	50 (30.49)
5	94 (57.32)	2 (1.22)	2 (1.22)	6 (3.66)	12 (7.32)	48 (29.27)
6	95 (57.93)	1 (0.61)	3 (1.83)	6 (3.66)	16 (9.76)	43 (26.22)
7	96 (58.54)	2 (1.22)	2 (1.22)	8 (4.88)	10 (6.10)	46 (28.05)
8	100(60.98)	1 (0.61)	5 (3.05)	5 (3.05)	9 (5.49)	44 (26.83)
9	82 (50.00)	1 (0.61)	2 (1.22)	4 (2.44)	11 (6.71)	64 (39.02)

**Key:** SDM, shared decision-making.

**Table A2:** Univariate analysis results.

Variable	Description	Coefficient
**Familism**		
Siblings ^c^	Number of siblings (0, 1, 2, 3, 4, …)	0.050 *
**Baseline characteristics**		
Age ^b^	0 = 44 to 55; 1 = 56 to 65; 2 = 66 to 75; 3 = 76+	0.027 *
Gender ^a^	0 = male; 1 = female	0.150 *
Schooling years ^b^	0 = 6−; 1 = 7 to 9; 2 = 9+	0.250
Marital status ^a^	0 = otherwise; 1 = married	0.250
Residence ^a^	0 = cities and towns; 1 = country areas	0.700
Household head ^a^	0 = No; 1 = Yes	0.144 *
Monthly income ^b^	0 = $152−; 1 = $152 to $454; 2 = $454+	0.691
**Personality**		
Agreeableness ^c^	In a negative description, 1 = Strongly Agree; 2 = Somewhat agree; 3 = Neutral; 4 = Somewhat disagree; 5 = Strongly Disagree In a positive description, 1 = Strongly Agree; 2 = Somewhat agree; 3 = Neutral; 4 = Somewhat disagree; 5 = Strongly Disagree	0.935
Conscientiousness ^c^		0.684
Extraversion ^c^		0.036 *
Neuroticism ^c^		0.419
Openness to experiences ^c^		0.000 *
**EOL care assessment**		
Staff approachableness ^c^	1 = Strongly Dislike; 2 = Somewhat Dislike 3 = Neutral 4 = Somewhat Like 5 = Strongly Like	0.621
Service quality ^c^		0.095 *
Clinician specialty ^c^		0.291
Service affordability ^c^		0.400
Service accessibility ^c^		0.672
**QoL**		
QoL self-evaluated score ^c^	From 1 = Very Bad to 10 = Very Good	0.244

**Key:** EOL, end-of-life; QoL, quality of care. **Notes:** ^a^ dichotomous; ^b^ ordinal categorical variable; ^c^ continuous variable; * *p* < 0.2.

**Table A3:** Brant test result for ordered logistic model.

	Chi2	*p* Value	Degree of Freedom
**Familism**			
Number of siblings	5.96	0.015	1
**Baseline characteristics**			
55–65 years old	0.34	0.557	1
66–75 years old	0.58	0.447	1
75+ years old	0.01	0.931	1
Female	0.72	0.396	1
Household head	0.01	0.916	1
**Personality**			
Extraversion	2.77	0.096	1
Openness to experiences	0.22	0.636	1
**EOL ^1^ care assessment**			
Service quality	0.38	0.535	1
**All factors**	1.88	0.993	9

^1^: EOL, end-of-life.
